# Left ventricular lead implantation using intravascular ultrasound–guided wiring and anchor balloon technique for a challenging case with persistent left superior vena cava

**DOI:** 10.1016/j.hrcr.2023.10.018

**Published:** 2023-10-28

**Authors:** Yuki Tanaka, Tadafumi Nanbu, Izumi Yoshida, Akihiko Yotsukura, Masayuki Sakurai

**Affiliations:** Department of Cardiology, Hokko Memorial Hospital, Sapporo City, Japan

**Keywords:** PLSVC, CRT, IVUS, Anchor balloon, Challenging case

**Key Teaching Points**

• Achieving successful cardiac resynchronization therapy (CRT) implantation can be challenging, especially in cases involving coronary sinus (CS) anomaly.
• The advanced anchor balloon technique, in which we dilate an anchor balloon at the stenotic lesion, is effective for cannulation of the stenotic CS branch.
• Intravascular ultrasound–guided wiring enabled us to insert the wire into the target vein precisely, even though we could not visualize the CS branch with any other modalities. This technique may help reduce the dose of contrast media required for CRT implantation procedures.

## Introduction

Cardiac resynchronization therapy (CRT) provides benefit to patients with symptomatic heart failure, severe left ventricular (LV) systolic dysfunction, and LV dyssynchrony. However, the procedure is often difficult owing to the LV lead implantation. One of the reasons for the difficulty of LV lead implantation is anomaly of the coronary sinus (CS).^1^

## Case report

**Figure 1:**
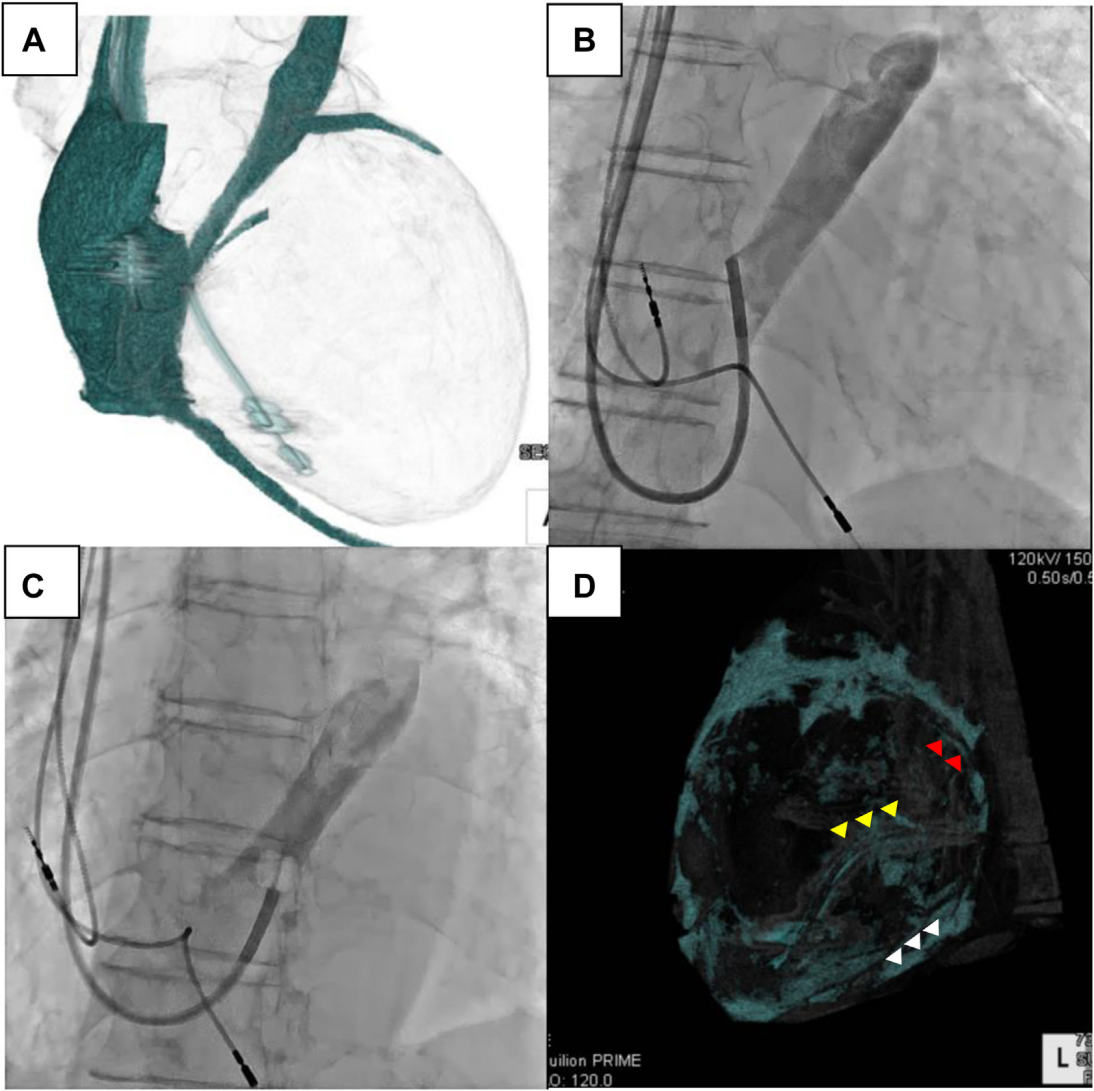
**A:** Contrast cardiac computed tomography (CT) image of anterior-posterior view showing persistent left superior vena cava and a coronary sinus (CS) without a lateral vein. CS branches are manually traced on a 2D image and reconstructed to a 3D image. **B:** Venography from the mid CS trunk. **C:** Occlusive venogram failed to visualize the CS branch. **D:** Noncontrast cardiac CT image using the fat description method of the left posterolateral view. Red arrowheads show the anterior intraventricular vein orifice. Yellow arrowheads show the right ventricular lead. White arrowheads show the middle cardiac vein.

A 73-year-old man with a history of heart failure due to dilated cardiomyopathy developed exertional breathlessness in his day-to-day life. He was hospitalized to upgrade his permanent pacemaker to a cardiac resynchronization pacemaker because of the heart failure owing to dilated cardiomyopathy. He had undergone catheter ablation for atrial fibrillation and common atrial flutter 3 years prior. Electrocardiography revealed a left bundle branch block, with a QRS duration of 224 ms. Echocardiography showed that the LV ejection fraction was 24%, the LV end-diastolic dimension was 85.6 mm, there was mild mitral regurgitation and moderate aortic regurgitation, and the septal-to-posterior-wall motion delay was 169 ms. Coronary angiography did not reveal the CS branch. Contrast-enhanced cardiac computed tomography (CT) revealed a persistent left superior vena cava (PLSVC) and a CS without a lateral vein (Figure 1A). An insertion of the LV lead into the anterior intraventricular vein (AIV) was planned to maximize the separation of the right ventricular and LV electrodes.

**Figure 2:**
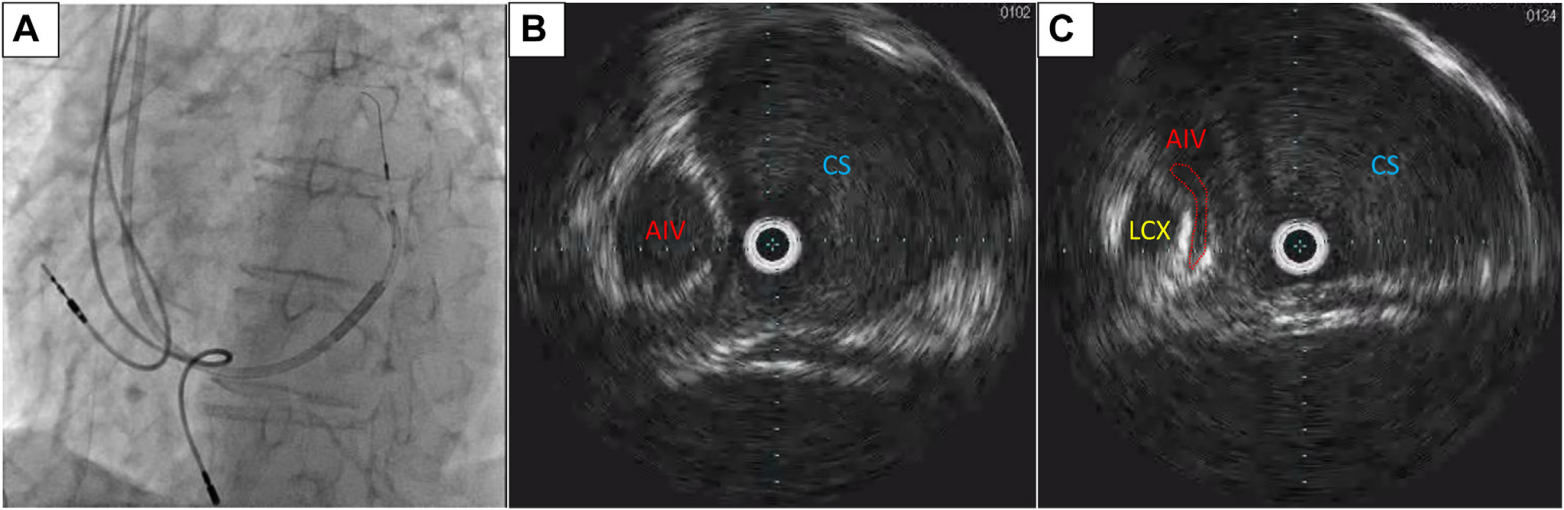
**A:** Fluoroscopy of intravascular ultrasound (IVUS) and guiding catheter at left anterior oblique view. **B:** IVUS image of the same level of (A). **C:** IVUS image close to anterior intraventricular vein (AIV). Note the AIV orifice is sandwiched between left circumflex (LCX) and coronary sinus (CS) trunk and shows eccentric narrowing.

**Figure 3:**
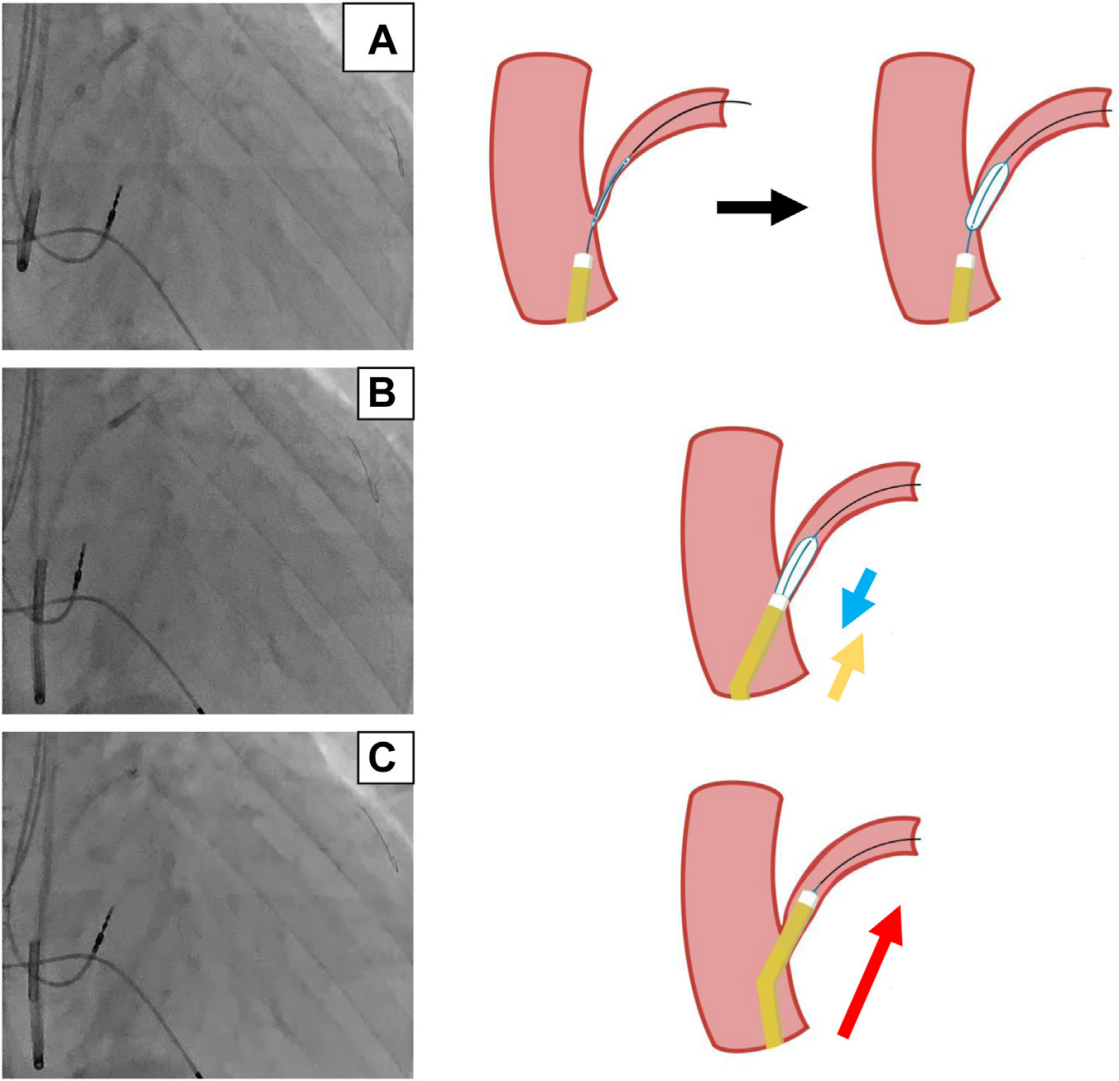
Advanced anchor balloon technique. **A:** The balloon was inflated at the anterior intraventricular vein (AIV) orifice as an anchor at the stenotic lesion. **B:** Balloon tail in contact with child catheter owing to gentle traction on the balloon and gentle forward pressure on the child catheter with continuous force. **C:** When the balloon was deflated, the child catheter was drawn automatically into the AIV, passing through the stenotic lesion.

The procedure was performed using a right subclavian vein approach. A 9F short sheath (Medtronic, Minneapolis, MN) was inserted into the right subclavian vein. A guiding catheter (Medtronic) was advanced into the main CS trunk using a 0.035-inch wire (Terumo, Tokyo, Japan). Multidirectional venography using a guiding catheter positioned in the mid CS trunk did not demonstrate AIV (Figure 1B). Moreover, the occlusive venography failed to visualize the CS branch because the balloon diameter was smaller than the CS trunk diameter (Figure 1C). Therefore, we decided to use intravascular ultrasound (IVUS) (Eagle Eye Platinum ST; Philips Volcano, CA) to detect the AIV orifice. An IVUS catheter was inserted into the CS using a mother-in-child catheter system. The IVUS catheter was placed close to the AIV orifice by rotating the child catheter, which had a 130-degree bend at the tip (Medtronic) (Figure 2A and 2B). A second 0.014-inch wire (Cruise; Asahi Intecc, Aichi, Japan) was inserted into the AIV under real-time guidance of the IVUS image. Simultaneously, the IVUS image revealed venous stenosis at the AIV orifice because it was sandwiched between the left circumflex artery and the CS trunk (Figure 2C). Since the child catheter could not be advanced to the AIV through the wire inserted into the distal AIV, we attempted to advance the child catheter using the anchor balloon technique with a 2.0 mm coronary balloon (Ryurei; Terumo).^2^ However, this attempt failed. The child catheter may not have passed through the AIV orifice owing to the venous stenosis. The balloon was then inflated at the AIV orifice and used as an anchor balloon at the point where the stenotic lesion was located (Figure 3A). Subsequently, we applied gentle traction to the balloon and gentle forward pressure to the child catheter so that the balloon tail came into contact with the child catheter tip, and traction and forward pressure was exerted (Figure 3B). The balloon was subsequently deflated. Consequently, the catheter was drawn into the AIV, and it passed through the stenotic lesion (Figure 3C). The LV lead advanced smoothly into the distal AIV because of the stability and strong backup force created by the child catheter that was inserted into the AIV. The pacing thresholds, sensing voltage, and impedance values were all within the acceptable ranges.

No complications such as a CS dissection or perforation occurred during or after the procedure. LV lead dislodgment and the loss of LV capture were not documented 3 months after the procedure. The QRS duration remained unchanged postoperatively. However, the chest radiography revealed an improvement in the cardiothoracic ratio from 64% to 55%. The patient’s symptoms markedly improved.

## Discussion

According to a previous report, the procedure success rate of CRT implantation was 89.2%–94.1%.^3^ The reasons most commonly cited for unsuccessful implantation include the inability to access the CS ostium, acute dislodgement or unstable lead position, and inability to obtain a distal lead position.

In this case, the first issue we encountered was how to pass the lead through the stenotic lesion. A technique using a coronary balloon as an anchor to facilitate the initial CS cannulation and LV lead placement has also been reported.^4^^,^^5^ The anchor balloon technique facilitates the catheter cannulation with a strong backup force and corrects co-axiality. However, this technique does not work well for stenotic veins, because the outer diameter of the catheter is larger than the venous luminal diameter. To solve this problem, we dilated the balloon at the stenotic AIV orifice and applied forward force to the catheter. As a result of the continuous force, when the balloon was deflated, it became smaller at the proximal site, while the distal site remained anchored. Consequently, a child catheter was drawn into the gap between the balloon and vessel. The child catheter was then automatically advanced into the AIV, passing through the stenotic lesion following balloon deflation. This advanced anchor balloon technique is useful for cannulation of the stenotic CS branch.

The second issue was the detection of an orifice that could not be visualized using other modalities. In the dilated CS, the IVUS scan revealed the precise level, direction, and proximity to the orifice, and IVUS-guided wiring enabled us to insert the wire to the target vein precisely. Furthermore, stenosis was observed in the AIV orifice. This finding was useful for the later cannulation of the child catheter.

Renal dysfunction is one of the most representative comorbidities in patients with heart failure. Although renal dysfunction is a risk factor of worsened prognosis,^6^ CRT benefits patients with heart failure regardless of renal dysfunction.^7^ However, patients with renal dysfunction often carry the risk of contrast-induced nephropathy. As such, IVUS-guided wiring has an advantage for LV lead implantation. Using this wiring technique, we do not need visualization of the target branch with venography. In other words, we could reduce the dose of the contrast media for CRT implantation. In addition, a method for visualizing coronary venous anatomy on noncontrast CT has been reported.^8^ The method uses the epicardial fat surrounding the vessels to create tissue contrast and render the vessel outlines clearly visible; using standard 3D workstations, the image data are inverted, and the window level and width are set to approximately −300 and 600 Hounsfield units, respectively. Using appropriate CT value extraction, 74% of the coronary venous branches relevant to CRT could be identified by CT without intravenous contrast,^8^ and in PLSVC cases, the dilated CS trunk is dilated and thus easier to visualize. In our patient, we identified the AIV on noncontrast CT (Figure 1D). In the future, a combination of the noncontrast CT method and IVUS-guided wiring may enable successful contrast-free CRT lead implantation for patients with severe renal dysfunction.

## Conclusion

In conclusion, the advanced anchor balloon technique and the IVUS-guided wiring are effective methods that can be used to implant an LV lead in challenging PLSVC cases. IVUS-guided wiring has another advantage for CRT implantation in that it does not require contrast media for LV lead implantation.

## Disclosures

The authors have no conflicts of interest to disclose.

## References

[1] Worley S.J., Gohn D.C., Pulliam R.W. (2008). Interventional approach to CRT in a patient with drainage of the superior vena cava into the coronary sinus. Pacing Clin Electrophysiol.

[2] Fujita S., Tamai H., Kyo E. (2003). New technique for superior guiding catheter support during advancement of a balloon in coronary angioplasty: the anchor technique. Catheter Cardiovasc Interv.

[3] Leon A.R., Abraham W.T., Curtis A.B. (2005). Safety of transvenous cardiac resynchronization system implantation in patients with chronic heart failure: combined results of over 2,000 patients from a multicenter study program. J Am Coll Cardiol.

[4] Worley S.J. (2009). How to use balloons as anchors to facilitate cannulation of the coronary sinus left ventricular lead placement and to regain lost coronary sinus or target vein access. Heart Rhythm.

[5] Narikawa M., Kiyokuni M., Taguchi Y. (2021). Successful implantation of left ventricular lead for a cardiac resynchronization therapy defibrillator through a persistent left superior vena cava using the anchor balloon technique. J Cardiol Cases.

[6] McAlister F.A., Ezekowitz J., Tonelli M., Armstrong P.W. (2004). Renal insufficiency and heart failure: prognostic and therapeutic implications from a prospective cohort study. Circulation.

[7] Daimee U.A., Moss A.J., Biton Y. (2015). Long-term outcomes with cardiac resynchronization therapy in patients with mild heart failure with moderate renal dysfunction. Circ Heart Fail.

[8] Tokudome D., Kowase S., Fujihara N. (2023). Efficacy of novel method of coronary vein description with non-contrast computed tomography. Japanese Journal of Electrocardiology.

